# Effect of COVID-19 on Glycemic Control, Insulin Resistance, and pH in Elderly Patients With Type 2 Diabetes

**DOI:** 10.7759/cureus.35390

**Published:** 2023-02-23

**Authors:** Saad Alshammari, Abdulrazaq S AlMasoudi, Aeshah H AlBuhayri, Hind M AlAtwi, Shemah S AlHwiti, Hind M Alaidi, Abdullah M Alshehri, Nouf A Alanazi, Ahmed Aljabri, Mohammed M Al-Gayyar

**Affiliations:** 1 PharmD Program, University of Tabuk, Tabuk, SAU; 2 Nursing, King Fahd Specialist Hospital, Tabuk, SAU; 3 Medicine, King Fahd Specialist Hospital, Tabuk, SAU; 4 Pharmacy, University of Tabuk, Faculty of Pharmacy, Tabuk, SAU; 5 Pharmacy, King Abdulaziz University Faculty of Pharmacy, Jeddah, SAU; 6 Pharmaceutical Chemistry, University of Tabuk, Faculty of Pharmacy, Tabuk, SAU; 7 Biochemistry, Mansoura University Faculty of Pharmacy, Mansoura, EGY

**Keywords:** metabolic score for insulin resistance (mets-ir), triglyceride to high-density lipoprotein cholesterol ratio (tg/hdl-c), triglyceride glucose-bmi (tyg-bmi), triglyceride-glucose index (tyg), covid-19

## Abstract

Background

The coronavirus disease of 2019 (COVID-19) was spread all over the world, while diabetes mellitus (DM) remains the most prevalent chronic disease worldwide.

Aims

This study aims to investigate the effect of COVID-19 on glycemic control, insulin resistance (IR), and pH in elderly patients with type 2 diabetes.

Methods

A retrospective study was conducted on patients with type 2 DM who were diagnosed with COVID-19 infection in the central hospitals of the Tabuk region. Patient data were collected from September 2021 to August 2022. Four non-insulin-based insulin resistance indexes were calculated for patients: the triglyceride-glucose (TyG) index, the triglyceride glucose-body mass index (TyG-BMI) index, the triglyceride to high-density lipoprotein cholesterol (TG/HDL) ratio, and the metabolic score for insulin resistance (METS-IR).

Results

Patients showed increased serum fasting glucose and blood HbA1c associated with a high TyG index, TyG-BMI index, TG/HDL ratio, and METS-IR as compared with results before COVID-19. Moreover, during COVID-19, patients revealed a reduction in pH, associated with a reduction in cBase and bicarbonate, and an elevation in PaCO_2_ as compared with their results before COVID-19. After complete remission, all patients’ results turn back to their level before COVID-19.

Conclusions

Patients with type 2 DM who catch the COVID-19 infection suffer from dysregulation of glycemic control and elevated insulin resistance associated with a significant reduction in their pH.

## Introduction

In the beginning of 2020, the coronavirus disease 2019 (COVID-19) had spread worldwide, endangering millions of lives. Several measures were put in place to stop the new coronavirus from spreading, including social isolation, which led to limitations on employment and physical activity and worsened quality of life. People were confined to their houses with high stress levels, low physical activity, and declining health patterns [1]. Moreover, the undertaken restrictive measures limit the person’s ability to access health services, supplies, and medicines, leading to great disturbances in metabolic control. Otherwise, the availability of high spare time at home enhanced better eating habits, physical activity, and weight loss to attenuate COVID-19 complications in those people at high risk of developing the disease’s complications [2].

Out of many observational studies and clinical studies, many risk factors that lead to poor COVID-19 outcomes have been identified. One of these famous risk factors is diabetes mellitus (DM), one of the most prevalent chronic diseases worldwide. DM occurs frequently with other comorbidities [3]. DM is reported to be the second most prevalent comorbidity in COVID-19 patients, just after hypertension. These studies reported the association between DM and COVID-19 severity, poor prognosis, and even mortality [4].

DM leads to many molecular changes that enhance the pathogenicity of COVID-19 and make DM patients vulnerable to COVID-19. DM significantly reduced phagocytic activity and neutrophil chemotaxis, resulting in diminished T cell function and innate and adaptive immunity deactivation. In addition, DM significantly elevated angiotensin-converting enzyme-2 (ACE2) levels [5]. Elevated ACE2 expression in human lung alveolar cells and other various body tissues has a high binding affinity with COVID-19, which works as an entry receptor for the virus and reduces its clearance [6]. Moreover, increased glucose concentration directly elevated the ability of COVID-19 to replicate in the body, which is associated with the deactivation of the immune system and the expression of inflammatory mediators, which causes a possible lethal complication. Compared to non-diabetic patients with the COVID-19 infection, those with diabetes mellitus experienced greater reductions in oxygenation status and disturbances in blood minerals [3]. Therefore, treating diabetic patients who have a COVID-19 infection requires more attention.

Insulin resistance (IR) is defined as any decrease in tissue response to the stimulatory effects of insulin, resulting in an imbalance in glucose metabolism, oxidative stress, and an inflammatory reaction [7]. Although there were some previous measures for controlling IR by controlling insulin concentrations in the body, the process was both complicated and costly. Consequently, researchers have adopted the triglyceride and glucose (TyG) index [8], the triglyceride glucose-body mass index (TyG-BMI) [9], the triglyceride to high-density lipoprotein cholesterol (TG/HDL-C) ratio [10], and the Metabolic Score for Insulin Resistance (METS-IR) [11] as simple and reliable substitutes for IR. There is a lack of evidence on the impact of COVID-19 on glycemic control in Saudi patients. Therefore, we aimed to investigate the effect of COVID-19 in patients with DM through the discovery of the direct effect of COVID-19 infection on glycemic control. In addition, we aimed to measure the effect of COVID-19 on the non-insulin-based insulin resistance indexes, TyG, TyG-BMI, TG/HDL-C, and METS-IR, in elderly patients with type 2 diabetes.

## Materials and methods

Study design and population

This was a retrospective study conducted between September 2021 and August 2022 on elderly patients with type-2 DM who were diagnosed with COVID-19 infection in the central hospitals of Tabuk region, Northwest Saudi Arabia. The Institutional Review Board at the General Directorate of Health Affairs in Tabuk Region approved the study protocol under registration number H-07-TU-077. All patient data were collected while ensuring the privacy of patients. The inclusion criteria were patients diagnosed with type 2 DM for at least six months, with an age equal to or greater than 45 years, infected with COVID-19, and who visited the clinics after completing COVID-19 treatment. The exclusion criteria consist of active malignancy, any anti-diabetic medication change in the last three months, severe infection or surgery after the last follow-up visit, and decompensated liver disease or heart failure.

Data collection

After receiving proper training, data were obtained from patient files. The files were collected, and physicians’ notes were reviewed to confirm the diagnosis of DM as well as those of patients who had COVID-19. The collected data included demographic information, comorbid diseases (hypertension, hyperlipidemia, cardiac diseases, chronic kidney disease, and respiratory disorders), current anti-diabetic medications, and clinical laboratory data.

Study outcomes

The effect of COVID-19 on glycemia control was measured by evaluating and comparing the fasting blood glucose level and HbA1c before, during, and after complete recovery from COVID-19 infection. The impact of COVID-19 on the non-insulin-based insulin resistance indexes as well as the pH was evaluated in the same manner. The effect of gender differences on the studied outcomes was also evaluated.

Evaluation of non-insulin-based insulin resistance index

BMI was calculated as weight (kg) divided by the square of height (m^2^):

TyG = Ln [TG (mg/dL) × FPG (mg/dL) ÷ 2] [8].

TyG-BMI = TyG × BMI (kg/m^2^) [9].

TG/HDL-C = TG (mg/dl) ÷ HDL-C (mg/dl) [10].

METS-IR = Ln [(2 × FPG (mg/dl)) + TG (mg/dl)] × BMI (kg/m^2^) ÷ Ln [HDL-C (mg/dl)] [11].

Statistical analysis

For quantitative variables, the mean ± standard error was used. The normality of the sample distribution was tested with the Kolmogorov-Smirnov (K-S) test. For comparison between groups, repeated measures ANOVA was used, followed by the Tukey HSD as a post hoc test. Statistical computations were done on a personal computer using the computer software SPSS version 20 (Chicago, IL, USA). Statistical significance was predefined as P < 0.05.

## Results

Patients’ characteristics

A total of 65 patients met the inclusion criteria, and none were excluded. The patients’ characteristics are summarized in Table 1. The patients' average age was 61.46 ± 1.63 years. Out of the included patients, about 54% were female and 46.15% were male. About 46% of the patients did not suffer from any other chronic diseases. The most comorbid diseases are hypertension (53.85%), hyperlipidemia (50.77%), cardiovascular diseases (43.08%), chronic kidney diseases (21.54%), respiratory diseases (9.23%), and parkinsonism (7.69%). Regarding the types of anti-diabetic drugs used, patients used different types of drugs. The most frequently used medications were insulin (28.12%), metformin (26.56%), and sulfonylurea (27.69%) (Table 1).

**Table 1:** Patients’ characteristics.

Parameter		Results
Age (years)		61.46 ± 1.63
Gender	Male	30 (46.15%)
	Female	35 (53.85%)
Comorbid diseases	No other comorbid diseases	30 (46.15%)
	Hypertension	35 (53.85%)
	Hyperlipidemia	33 (50.77%)
	Cardiovascular diseases	28 (43.08%)
	Chronic kidney diseases	14 (21.54%)
	Respiratory diseases	6 (9.23%)
	Parkinsonism	5 (7.69%)
Antidiabetic drugs	Insulin	18 (28.12%)
	Metformin	17 (26.56%)
	Sulfonylurea	18 (27.69%)
	Dipeptidyl peptidase-4 inhibitors	17 (26.15%)
	Insulin + metformin	13 (20.00%)
	Insulin + sulfonylurea	4 (6.15%)
	Metformin + dipeptidyl peptidase-4 inhibitors	4 (6.15%)
	Metformin + sulfonylurea	3 (4.62%)

Effect of COVID-19 on glycemic control in elderly patients with type 2 diabetes

Investigation of patients with type 2 DM revealed dysregulation of glycemic control after infection with COVID-19. Patients showed an increase in serum fasting glucose and blood HbA1c of 241.95 ± 10.57 mg/dl and 10.06 ± 0.37%, respectively, as compared with the results before COVID-19 infection (180.28 ± 6.86 mg/dl and 7.91 ± 0.24%, respectively). After complete remission from COVID-19 infection, patients showed significant reductions in serum fasting glucose and blood HbA1c, 192.89 ± 8.27 mg/dl and 8.35 ± 0.29%, respectively, as compared with their results during COVID-19 infection (Table 2).

**Table 2:** Effect of COVID-19 on glycemic control in elderly patients with type 2 diabetes. *Significant difference as compared with patients before COVID-19 infection at p<0.05. ^#^Significant difference as compared with patients during COVID-19 infection at p<0.05.

Parameter	Results (mean ± SE)			P-value of repeated measures ANOVA
	Before COVID-19	During COVID-19	After COVID-19	
Fasting glucose (mg/dl)	180.28 ± 6.86	241.95 ± 10.57*	192.89 ± 8.27^#^	p<0.01
HbA1c (%)	7.91 ± 0.24	10.06 ± 0.37*	8.35 ± 0.29^#^	p<0.01

Effect of COVID-19 on the non-insulin-based insulin resistance index in elderly patients with type 2 diabetes

After COVID-19 infection, elderly patients with type 2 DM suffered from significant increases in TyG, TyG-BMI, TG/HDL-C, and METS-IR as compared with the results of patients before COVID-19 infection. However, after the COVID-19 infection, all the insulin resistance indexes returned to their levels before the COVID-19 infection (Table 3).

**Table 3:** Effect of COVID-19 on glycemic control in elderly patients with type 2 diabetes. *Significant difference as compared with patients before COVID-19 infection at p<0.05. ^#^Significant difference as compared with patients during COVID-19 infection at p<0.05.

Parameter	Results (mean ± SE)			P-value of repeated measures ANOVA
	Before COVID-19	During COVID-19	After COVID-19	
TyG	9.52 ± 0.041	9.95 ± 0.052*	9.68 ± 0.047*^#^	P<0.001
TyG-BMI	312.71 ± 7.66	326.80 ± 7.96*	318.13 ± 7.85*^#^	P<0.001
TG/HDL-C	3.85 ± 0.085	4.84 ± 0.119*	4.37 ± 0.103*^#^	P<0.001
METS-IR	54.93 ± 1.33	58.04 ± 1.38*	55.91 ± 1.34*^#^	P<0.001

Effect of COVID-19 on pH in elderly patients with type 2 diabetes

Patients showed a reduction in pH during the COVID-19 infection, which was increased to the levels before the infection after complete remission. In addition, there was a significant reduction in both cBase and bicarbonate during COVID-19 infection, which was associated with a significant elevation in PaCO_2_ as compared with their results before COVID-19 infection. After complete remission, patients showed a significant increase in cBase and bicarbonate as well as a reduction in PaCO_2_ as compared with their results during COVID-19 infection (Table 4).

**Table 4:** Effect of COVID-19 on pH in elderly patients with type 2 diabetes. *Significant difference as compared with patients before COVID-19 infection at p<0.05. ^#^Significant difference as compared with patients during COVID-19 infection at p<0.05.

Parameter	Results (mean ± SE)			P-value of repeated measures ANOVA
	Before COVID-19	During COVID-19	After COVID-19	
pH	7.37 ± 0.02	7.31 ± 0.02*	7.42 ± 0.01*^#^	p<0.01
cBase (mEq/L)	–0.41 ± 0.31	–1.87 ± 0.39*	0.48 ± 0.36*	p<0.01
Bicarbonate (mEq/L)	26.12 ± 0.42	24.04 ± 0.39*	25.98 ± 0.39*	p<0.01
PaCO_2_ (mm Hg)	35.03 ± 0.51	40.97 ± 1.29*	37.95 ± 0.70*^#^	p<0.05

Effect of gender on patients’ outcomes during COVID-19 infection

After the classification of patients according to gender, we discovered that there were no significant differences between male and female patients in all measured parameters during COVID-19 infection (Table 5).

**Table 5:** Effect of gender on patients results during COVID-19 infection.

Parameter	Male patients (n = 30)	Female patients (n = 35)	P-value
Glucose (mg/dl), mean ± SE	249.83 ± 15.44	235.17 ± 14.18	0.24
HbA1c (%), mean ± SE	10.33 ± 0.54	9.82 ± 049	0.24
TyG, mean ± SE	10.00 ± 0.08	9.91 ± 0.06	0.18
TyG-BMI, mean ± SE	326.86 ± 10.93	326.76 ± 11.59	0.50
TG/HDL-C, mean ± SE	4.93 ± 0.16	4.75 ± 0.17	0.23
METS-IR, mean ± SE	58.09 ± 2.02	58.00 ± 1.93	0.49
pH, mean ± SE	7.32 ± 0.025	7.29 ± 0.024	0.17
cBase (mEq/L), mean ± SE	–1.44 ± 0.53	–2.24 ± 0.56	0.15
Bicarbonate (mEq/L), mean ± SE	24.25 ± 0.62	23.85 ± 0.48	0.30
PaCO_2_ (mm Hg), mean ± SE	43.22 ± 2.24	39.04 ± 1.30	0.06

## Discussion

When the COVID-19 pandemic first started, many problems arose with the evolution of the restrictions on access to healthcare, leading to poor outcomes. One of the most common diseases worldwide is diabetes. Several studies reported a decline in glycemic control among COVID-19 patients during lockdown [12,13]. Early reports arising after the COVID-19 pandemic illustrated the great risk of severe COVID-19 infection among diabetic patients [14]. Recent reports illustrated the association between high HbA1c levels and inflammation, hypercoagulability, and low oxygen saturation [15]. Therefore, epidemiological data confirm a 3.5-fold elevation of the risk of COVID-19-related death in patients with diabetes [16]. All these results pointed out the need for great attention to patients with diabetes during the COVID-19 pandemic, with great emphasis on the maintenance of optimal glycemic control as an important component required for overall health [17]. Therefore, we conducted this study on elderly patients with type 2 diabetes. We followed the patients’ outcomes before, during, and after the complete remission of COVID-19 in the hospitals of Northwest Saudi Arabia. Compared to results prior to COVID-19 infection, our results demonstrated an increase in serum fasting glucose and blood HbA1c, which were then significantly reduced after complete remission to their levels prior to COVID-19 infection.

Some previous studies reported the dysregulation of glycemic control associated with weight gain in diabetic patients during the COVID-19 pandemic [18]. The dysregulation of glycemic control could be attributed to increased consumption of sugary foods and snacks with decreased physical activity and limited access to health services during the pandemic, resulting in changes in body composition [19]. Part of these studies illustrated the decline in the physical activity of the lower extremities as a reduction in the number of daily steps and an increase in sedentary time [18]. Moreover, social distance and staying at home could lead to lifestyle and behavioral changes that could affect the psychological status of the patients. In addition, the anxiety that arises among many elderly patients with type 2 diabetes could produce adverse effects on the patient’s health. Psychological factors, especially anxiety, are linked to the pathology of chronic diseases due to the hypersensitivity of the sympathetic nervous system, with subsequent effects on glycemic control, insulin resistance, and the immune system [20].

Type 2 DM is an inflammatory disease characterized by endothelial damage, which is already present before the clinical diagnosis of diabetes, leading to insulin resistance and a poor outcome in patients with COVID-19 [21]. Insulin resistance is the major mechanism of hyperglycemia in diabetic patients, especially COVID-19 patients. Insulin resistance enhances the expression of ACE2 receptors, which promotes the entry of COVID-19 into the host cell, resulting in an increase in viral load and direct pancreatic damage caused by the virus [22]. Insulin resistance in type 2 diabetic patients leads to impaired metabolic and cardiovascular homeostasis, which in turn causes inflammation and immune suppressant effects, resulting in an increase in the severity of COVID-19 in patients. Therefore, we measured four different non-insulin-based insulin resistance indexes: TyG, TyG-BMI, TG/HDL-C, and METS-IR. We discovered a significant increase in the insulin resistance indices in elderly patients with type 2 diabetes during the COVID-19 infection, which was reduced after complete remission.

Insulin resistance is linked to overweight and obesity. Studies that measured body composition illustrated elevated body fat mass and reduced body skeletal muscle mass. All these studies lead to the hypothesis of increased sensibility to sarcopenic obesity in diabetic patients, which is associated with enhanced deterioration of function and increased risks of cardiometabolic disease and mortality [23]. It should be taken into consideration that we conducted our study on elderly patients and that sarcopenic obesity increases with age [24]. All these effects can lead to insulin resistance.

Acidosis is defined as any reduction in the blood pH. It occurs for many reasons. Respiratory acidosis is caused by the inability of the lungs to remove carbon dioxide. Metabolic acidosis takes place due to a lack of bicarbonate, enhanced production of acid, and the inability of the kidneys to remove acids [25]. Metabolic acidosis in COVID‐19 patients has been widely reported. The COVID-19 infection not only exacerbates acidosis in diabetic patients but also directly causes it [26]. Moreover, COVID‐19 promotes respiratory acidosis among critically ill patients [27]. As a way to quickly reverse acute acidemia, the previous pole of therapy placed great assertiveness on intravenous sodium bicarbonate, which significantly reduces the primary composite outcome and mortality in patients with acute kidney injury [28]. We observed a decrease in pH in elderly patients with type 2 diabetes, which was reversed following complete remission from COVID-19. As the glucose level rises during any infection and COVID-19 affects the lungs, this results in a high pH. It has been reported that COVID-19 patients with acidosis exhibit a 37% overall mortality rate, which is higher than that of COVID-19 diabetic patients without acidosis [29]. Severe cases of COVID-19 promote acute respiratory distress syndrome, metabolic acidosis, and multiple organ dysfunction syndromes, among other complications [30]. In parallel, acidosis enhances COVID-19 infection by increasing cell membrane ACE2 expression. Therefore, acidosis promotes further infection and difficulty in recovery from COVID-19 [28].

Although the data were collected from many public hospitals in the Northwest region of Saudi Arabia, the only limitation present was the collection of patient data from only one region of Saudi Arabia, limiting the number of patients enrolled.

## Conclusions

Patients with type 2 DM, who catch the COVID-19 infection suffer from dysregulation of glycemic control, as revealed by a severe increase in fasting blood glucose and HbA1c. In addition, they suffered from a significant increase in four indices of non-insulin-based insulin resistance. Moreover, elderly patients with type 2 DM who catch a COVID-19 infection suffer from a reduction in their pH. There are no differences between male and female patients in all measured parameters. After complete remission of COVID-19, the glycemic control and pH return to their normal levels before the COVID-19 infection.
